# Fast Screening of Diol Impurities in Methoxy Poly(Ethylene Glycol)s (mPEG)s by Liquid Chromatography on Monolithic Silica Rods

**DOI:** 10.3390/polym10121395

**Published:** 2018-12-16

**Authors:** Michaela Brunzel, Tobias C. Majdanski, Jürgen Vitz, Ivo Nischang, Ulrich S. Schubert

**Affiliations:** 1 Laboratory of Organic and Macromolecular Chemistry (IOMC), Friedrich Schiller University Jena, Humboldtstr. 10, 07743 Jena, Germany; michaela.brunzel@uni-jena.de (M.B.); tobias.majdanski@kit.de (T.C.M.); j.vitz@uni-jena.de (J.V.); 2 Jena Center for Soft Matter (JCSM), Friedrich Schiller University Jena, Philosophenweg 7, 07743 Jena, Germany; 3 Center for Sepsis Control and Care (CSCC), Jena University Hospital, Am Klinikum 1, 07747 Jena, Germany

**Keywords:** anionic ring-opening polymerization, MALDI-TOF-MS, monolith, PEGdiol, pharmaceutical PEG, reversed-phase chromatography

## Abstract

The determination of diol impurities in methoxy poly(ethylene glycol)s (mPEG)s is of high importance, e.g., in the area of pharmaceutical applications, since mPEGs are considered the gold standard—based on properties of biocompatibility, stealth effect against the immune system, and well-established procedures used in PEGylation reactions. Herein, we communicate a straightforward and fast approach for the resolution of the PEGdiol impurities in mPEG products by liquid chromatography on reversed-phase monolithic silica-rods. Thus, we utilize fine, in-house prepared and narrow dispersity mPEGs (*Ð* ≤ 1.1) and commercial PEGdiol standards as a reference. Most efficient analysis of diol impurities becomes possible with reversed-phase liquid chromatography that results in selective elution of the PEGdiol from mPEG macromolecule populations in partition/adsorption mode. We do this by a minimum selectivity of the population of macromolecules characterizing the narrow molar mass distributions of mPEG. Control experiments with intentionally added water at the start of the well-controlled mPEG synthesis via the living anionic ring opening polymerization of ethylene oxide clearly reconciled the existence of PEGdiol impurity in chromatographed samples. The here-demonstrated methodology allows for the resolution of diol impurities of less than one percent in elution times of only a few minutes, confirmed by matrix-assisted laser desorption/ionization time-of-flight mass spectrometry (MALDI-TOF-MS) of the collected elution fractions. The unique combination of the open flow-through pore structure of the monolithic silica rods and resultant varying accessibility of C18-derivatized pore surfaces indicates beneficial properties for robust and end-group-specific adsorption/partition liquid chromatography of synthetic macromolecules.

## 1. Introduction

One of the most important pharmaceutically relevant polymers and as well the gold standard in medicine for a stealth effect by the PEGylation reaction is the well-established mPEG, which is also approved by the Food and Drug Administration (FDA) for a range of applications [1,2,3,4]. However, a well-known problem of outmost significance is the existence of PEGdiol impurities, which lead to cross-linking of proteins or other pharmaceutical components of interest, thus resulting in ill-defined species in PEGylation reactions [3]. 

**Figure 1:**
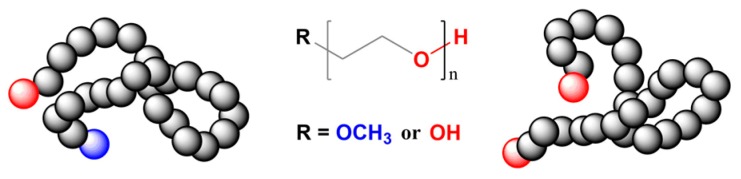
Schematic representation of an α-methoxy poly(ethylene glycol) (mPEG) with an ω-hydroxyl group and the corresponding PEGdiol.

The PEGdiol has very similar physicochemical properties compared to the mPEG (Figure 1) and, hence, it is difficult to distinguish between the two. Approaches from the literature used to address this problem include, e.g., nuclear magnetic resonance (NMR) spectroscopy [5,6,7], mass spectrometry (MS) [8], and last but not least the potential application of liquid chromatography [9,10,11,12], often hyphenated with suitable detection technology, e.g., evaporative light scattering detection (ELSD), MS, etc. Common analytical approaches in polymer science such as matrix-assisted laser desorption ionization mass spectrometry (MALDI-MS) and NMR alone have some inherent drawbacks. MALDI-MS of mixtures of different species provides difficulties due to the well-known mass discrimination as well as the different ionization efficiencies for mPEG vs. PEG. NMR is also not a very sensitive and precise method for such a purpose since the integrated backbone signal compared to the end-group largely increases. These aspects make liquid chromatography a highly interesting field of research, particularly in combination with orthogonal detection technology used for identification purposes.

According to the literature, the chromatography of synthetic macromolecules may be classified in three modes: size exclusion chromatography (SEC), liquid chromatography under critical conditions (LCCC), and liquid adsorption chromatography (LAC) [13]. LCCC as an elution mode may offer macromolecule chain length independent elution, i.e., being determined by the backbone respectively, end group only [14,15,16,17]. Fundamentally, however, the influence of the methoxy α-end in mPEG, as opposed to that of the hydroxyl, implies very limited differences in the overall hydrophobic/hydrophilic properties of PEG macromolecules (Figure 1), making straightforward resolution procedures based on liquid chromatography a challenging and so far unreported endeavor.

To the best of our knowledge, there has been no overarching approach, allowing for a straightforward resolution of PEGdiol impurities in mPEG over an extended molar mass range. If such possibility is indicated, then only for a very limited molar mass range far below 1000 g mol^−1^ of model compounds [18,19] or a single molar mass example [5,7,11]. The patent literature on this issue suggests that chromatographic resolution may become possible by the modification of the hydroxyl-terminal chain end(s) with groups of pronounced, e.g., hydrophobic character which results in functionalized ω-ends or functional α- and ω-chain ends that may show interaction with the particularly chosen chromatographic sorbents [20]. The efficiency of such labelling reactions is a further issue of consideration.

To address the quest for analyzing a very tiny difference in the end-group character of pharmaceutical mPEG as opposed to that of the PEGdiol in the relevant molar mass range by liquid chromatography, we employ C18-derivatized monolithic silica rods and disclose their potential for the above-mentioned problem. The high porosity silica-based monolithic materials are characterized by a macroporous, interconnected flow-through pore structure, confined by a continuous permanently mesoporous skeleton. Such stationary phase offers the highly efficient and retention-insensitive performance in the separation of small solutes [21]. Here, we investigate on their suitability for liquid chromatography of synthetic water-soluble and biocompatible macromolecules.

## 2. Materials and Methods

Chromatographic measurements were performed using an adapted Agilent Technologies 1200 series chromatographic system from Polymer Standards Service GmbH (PSS, Mainz, Germany) comprising a column oven and an evaporative light scattering detector (ELSD) operated with nitrogen as the carrier gas. Elutions of commercially available PEGdiols (PSS, Mainz, Germany and Polymer Laboratories, Shropshire, UK) and in-house-prepared mPEGs were performed on a Chromolith^®^ HighResolution RP-18 endcapped silica monolithic column, obtained as a research sample from Merck KgaA (Darmstadt, Germany). The length of the column was 100 mm and its inner diameter was 4.6 mm. The material features a high porosity of larger 80% with macropores of 1.1 µm in size, a mesopore size of 15 nm, and an internal surface area of 250 m^2^ g^−1^.

The molar mass (number-average, *M*_n_, as well as weight-average, *M*_w_) of the in-house prepared mPEGs was determined via size exclusion chromatography, as well indicating narrow dispersities of *Ð* ≤ 1.1 (Table S1 of the Supporting Information).

All samples were prepared at concentrations between 0.1 and 2.0 mg mL^−1^ by dissolving them in the respective mobile phase used for chromatographic experiments. Further details concerning the synthesis of the mPEGs, chromatographic equipment, measurements, and used chemicals are detailed in the Supporting Information.

## 3. Results and Discussion

**Figure 2:**
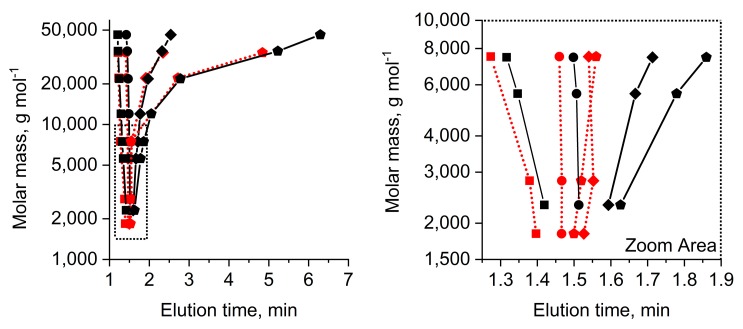
Molar mass (*M*_n_) elution time plot of PEGdiol (red symbols and red dotted connecting lines) and mPEG (black symbols and black connecting lines) indicating the transition between the size exclusion mode toward the adsorption/partition mode reached first for the larger molar masses. Symbols indicating percentage acetonitrile/water (%, *v*/*v*): 50/50 (squares), 42/58 (circles), 41/59 (diamonds), and 40/60 (pentagons). The zoom area clearly shows that the smaller mPEGs elute significantly later than diols at a mobile phase composition of 40/60 (pentagons). Mobile phase flow rate of 1 mL min^−1^.

Figure 2 shows selected results of elution studies of mPEGs and PEGdiols within a molar mass range of 1500 to 50,000 g mol^−1^. A complete study of the elution time vs. mobile phase composition for all the PEGs used in this study is shown in Figure S1.

It becomes clear that a mobile phase composition of 50/50 acetonitrile/water (%, *v*/*v*) results in elution patterns that are influenced by size exclusion effects, i.e., the largest PEGs elute first (black and red squares in Figure 2). Decreasing strength of the mobile phase eluent to 42% acetonitrile shows increased elution times for the larger molar mass PEGs, such that all PEGs elute at similar elution times with impractical selectivity (black and red circles) across all molar masses. This situation highlights the limited capability of a LCCC elution mode enabling distinction between the mPEG and PEGdiol since the difference of an α-hydroxyl as opposed to the α-methoxy is simply too small to enable clear critical adsorption conditions based on the end-group character (Figure 1). At eluent compositions containing only 41% and 40% acetonitrile, larger PEGs elute significantly later than the smaller ones, i.e., pronouncedly entering the partition/adsorption mode of the polymer backbone at molar masses above 10,000 g mol^−1^ (red and black diamonds and pentagons in Figure 2). It is also observable that the 7000–8000 g mol^−1^ mPEG/PEGdiols show the greatest differences in the partition/adsorption mode. Though it appears straightforward to judge on better separation ability for these molar masses (Figure S2), we also have to consider the increased elution width by longer residence times, in addition depending on the dispersity (Table S1). This originates from an increased selectivity for the polymeric species of each mPEG/PEGdiol population (Figure S2). The answer to this apparently observed selectivity issue can only be provided by carefully studying other functional end groups in PEG and will be in focus of our ongoing research. However, above 10,000 g mol^−1^ elution is entirely determined by molar mass-dependent adsorption/partition of the polymer backbone with a gradually decreasing contribution originating from the α-group identity that is clearly seen in the zoom area of Figure 2 for the smaller molar masses (< 10,000 g mol^−1^, pentagons).

**Figure 3:**
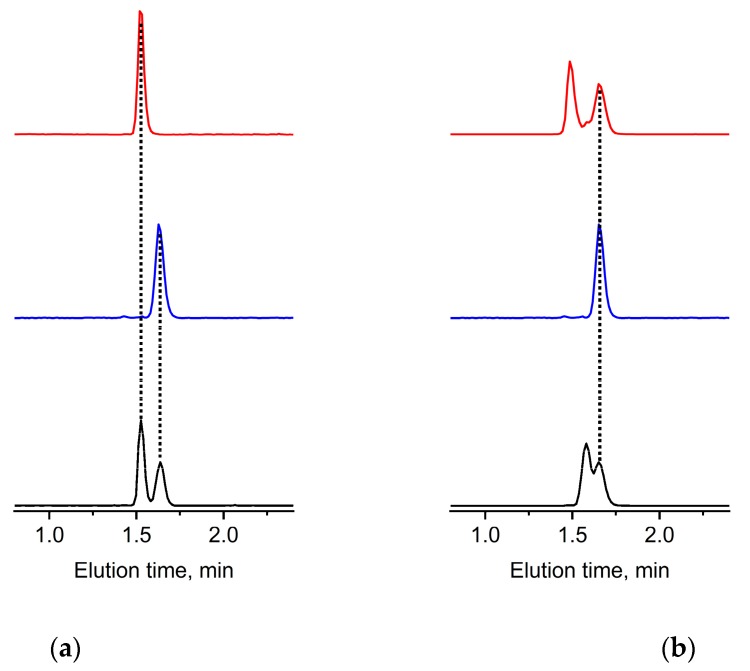
(**a**) PEGdiol (*M*_n_ = 1840 g mol^−1^, red trace), mPEG (*M*_n_ = 2300 g mol^−1^, blue trace), and their respective 50/50 (%, *v*/*v*) mixtures (black trace) and (**b**) mPEG (*M*_n_ = 2300 g mol^−1^, blue trace) together with *M*_n_ = 375 g mol^−1^ PEGdiol in a respective 50/50 (%, *v*/*v*) mixture (red trace), and *M*_n_ = 2800 g mol^−1^ PEGdiol in a respective 50/50 (%, *v*/*v*) mixture (black trace). Mobile phase flow rate of 1 mL min^−1^ and mobile phase composition of 40/60 acetonitrile/water (%, *v*/*v*).

At this point, partition and adsorption start dominating elution and, most significantly, the elution times of the population of species of the mPEG and PEGdiol start developing small but noticeable differences in elution times. In fact, Figure 2 highlights the only opportunity for the separation of mPEG and PEGdiol species of the similar molar masses, i.e., the partition/adsorption mode. While PEGdiols between 1000 and 10,000 g mol^−1^ still elute similarly, mPEGs show later elution (pentagons in Figure 2). This is shown by example separations of a mixture of a mPEG/PEGdiol pair of similar molar masses (Figure 3a). The results demonstrate that this mPEG example elutes as a narrow elution peak distinct from that of a similar PEGdiol. Larger 10,000 g mol^−1^ molar mass mPEGs elute similarly to the model diols of comparable molar mass (Figure 1). This situation is not surprising and originates from a lost selectivity with regard to the end group character at molar masses exceeding 10,000 g mol^−1^, where polymer chain adsorption strongly dominates elution. We note that if the diol has a distinct molar mass distribution it will still show up in the elugram, at larger molar masses possibly at later elution times than the mPEG.

To underpin the distinct selectivity between the mPEG and PEGdiol, we analyzed the mPEG and PEGdiol mixture example shown in Figure 3 by gradient liquid chromatography (Figure S3). The experiments showed the inherently expected dispersity of the PEGdiol standards and in-house-made mPEGs by a multiplicity of peaks for both the PEGdiol and mPEG sample. The gradient elution scenario does not improve distinction between the mPEG and PEGdiol (Figure S3) but allows chromatographic resolution of individual oligomeric species.

Based on varying initiation scenarios as well as kinetic arguments of the polymerization, the PEGdiol impurity is very unlikely having the same molar mass and distribution as the molar mass and its distribution for the desired mPEG. In a very recent work, by utilizing similar equipment and procedures for the preparation of diphenyl methane poly(ethylene glycol) (DPM-PEG), we observed the development of diol impurities being very sensitive to the kinetics of polymerization and solvents utilized for the anionic ring-opening polymerization (AROP) [22]. In general, the imposed different probability of initiation and kinetics of growing polymer chains with two possible monomer addition sites needs attention. Therefore, it is highly desirable to indicate whether smaller and, in particular, larger PEGdiol impurities, as suggested in the literature [11], are present in mPEG products. To mimic such conditions, we have studied mPEGs at the lower and the higher end of the molar mass range in mixtures with PEGdiols of smaller as well as larger molar mass. Figure 3b and Figure S4 show selected elugrams from such study.

It is clear that smaller molar mass diols are clearly resolved from that of the respective mPEG (Figure 3b and Figure S4, top red traces), however increasingly difficult to identify for larger molar mass examples (Figure 3b and Figure S4, bottom trace), though two eluting populations of species can be seen. The larger molar mass commercial PEGdiol example (Figure S4, bottom trace) elutes as two distinguishable fractions, originating from a bimodal molar mass distribution. However, these elute later than that of the mPEG. This is an inherent result of the PEGdiols elution based on polymer chain adsorption/partition by the macromolecules’ larger molar mass (Figure 2). The smaller elution fraction also shows some overlap with that of the mPEG. Notwithstanding, as opposed to the pure mPEG (Figure S4, middle trace), the PEGdiol is indicated by a clearly distinct elution pattern as seen in the chromatograms (Figure S4 bottom trace viz. the blue middle trace).

To indicate the principal suitability of an estimation of amounts of existing PEGdiols, we attempted experiments with varying concentrations of PEGdiol (*M*_n_ = 1840 g mol^−1^) to an example mPEG (*M*_n_ = 2300 g mol^−1^) of a fixed concentration (Figure 3a). A typical nonlinear dependence of the ELSD [11,23] even in the double logarithmic plot of peak height against solution concentration is apparent for experiments performed in triplicate (Figure S5b, black symbols). Notwithstanding, the repeatability of the measurements of the same sample solutions at different days, again performed in triplicate (Figure S5b, red symbols) justifies an estimation of concentrations and contents of PEGdiols reaching less than 1% in mPEG of a concentration of 1 mg mL^−1^.

**Figure 4:**
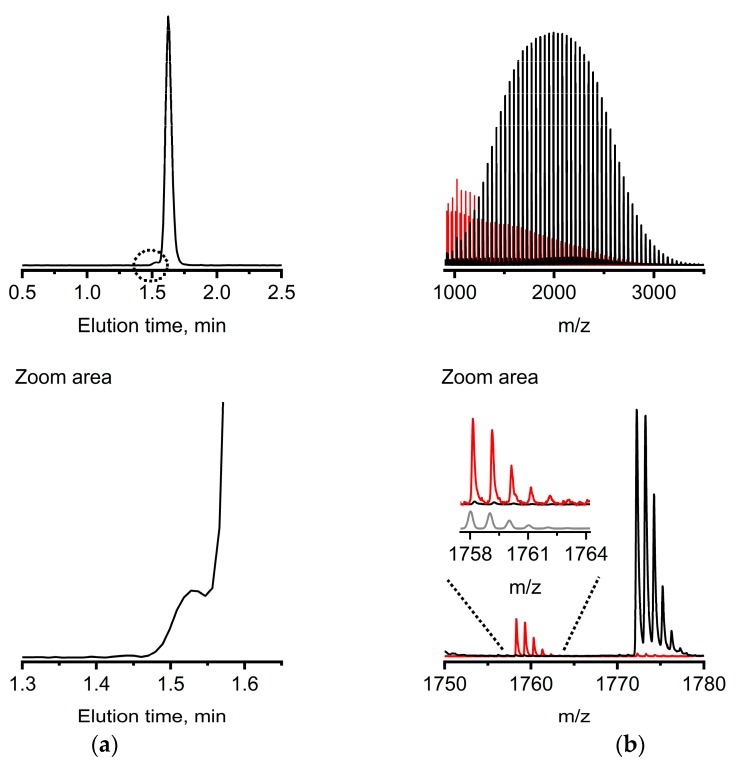
Application example of the developed methodology under the same chromatographic conditions as in Figure 3. (**a**) Elution trace of the 1 mg mL^−1^ anionic ring opening polymerization product containing potential diol impurity and (**b**) MALDI-TOF-MS of the collected small elution fraction identified as PEGdiol (shown in red) and major elution fraction identified as mPEG (shown in black). The inset in the right panel of (**b**) also contains the theoretical isotopic fragmentation pattern of PEGdiol shown in gray.

For a final demonstration of the opportunity developed by our approach, we analyzed a sample of mPEG that was prepared intentionally with a protic impurity, i.e., water, added at the start of the presumably living AROP. The MALDI-TOF-MS spectrum of this macromolecule population (Figure S6) indicates a diol impurity (Figure S6, inset). Unfortunately, MALDI-TOF-MS of inherently disperse polymer samples on its own is far away from being quantitative. This is due to well-known mass discrimination in disperse populations as well as the unknown ionization efficiencies for mPEG vs. PEGdiol in conjunction with the used matrix. Chromatographically, a diol impurity is indicated by a clear peak shoulder at smaller elution times in the chromatogram (Figure 4a). A carefully collected elution fraction (Figure 4a) was identified as PEGdiol impurity with a broad molar mass distribution from smaller 1000 m/z toward 3000 m/z by MALDI-TOF-MS (*M*_n_, _MALDI_ = 1500 g mol^−1^) as a positive orthogonal control (Figure 4b, red mass spectrum). The amount of PEGdiol was estimated as significant, with ca. 8% based on the brief response study with the ELSD (Figure S5). The molar mass of the mPEG without apparent features from the PEGdiol and based on MALDI-TOF-MS was estimated to *M*_n_, _MALDI_ = 2100 g mol^−1^. The apparently smaller molar mass of the PEGdiol impurity was observed recently in DPM-PEG preparations as well, affirmed by using different bases in the initial reaction mixture showing very poor initiation rates [22].

Summarizing from the above shown data, experimental conditions that do not result in selective elution of individual species characterizing the molar mass distribution (e.g., Figure S3), but molar mass distribution populations of mPEG and PEGdiol in fairly narrow elution peaks (e.g., Figure 3, Figure S4), appeared suitable for a quick resolution of PEGdiol impurities in mPEG in the pharmaceutically-relevant molar mass range (Figure 2). This is demonstrated for a practical application example utilizing AROP with a rather broadly distributed PEGdiol impurity (Figure 4).

Additional information from collected elution fractions investigated via MALDI-TOF-MS, including isotope pattern analysis (Figure 4b), were shown to ultimately answer the quest of analysis of the PEGdiol impurities in mPEG. Though, the example shown in Figure 4 provides no chromatographic baseline resolution since the diol has a rather broad molar mass distribution, further studies will focus on the tailoring of selectivity between the mPEG and PEGdiol populations by, e.g., adjusting the separation temperature. We finally note that during this project, over a timescale of approximately one year with at least 1000 injections, neither the elution time of the PEGs significantly changed, nor was a significant increase of backpressure observed by operating the column under retained conditions with molar masses reaching up to 50,000 g mol^−1^ (Figure 2).

## 4. Conclusions

We have demonstrated and explained how PEGdiol impurities in widely used mPEGs can be identified rapidly by fast liquid chromatography on C18-derivatized monolithic silica rods. The selection of this stationary phase was based on its particular properties characterized by an open, porous flow-through pore structure. This porous structure is distinct from those of packed beds, in which the macroporous structure as well is confined by direct contact between particles. A particular aspect for success of this approach, suitable for the pharmaceutically-relevant molar mass range, was the selection of chromatographic conditions that utilize the partition/adsorption mode, particularly conditions that characterize the start of the partition/adsorption regime, void of pronounced selectivity originating from the dispersity of macromolecule populations. Under these conditions, a minimal amount of band dispersion is apparent, since the readily low selectivity in the elution can be resolved chromatographically, therefore allowing for resolution of PEGdiol impurities. This also opens the door for removal of existing diol impurities by preparative chromatography utilizing monolithic silica rods. In forthcoming studies we will detail this opportunity for other, chromatographically less challenging PEG chain-termini prepared in our lab that are important for use in conjugation reactions or as components for creation of functional materials.

## Supplementary Materials

The following are available online at http://www.mdpi.com/2073-4360/10/12/1395/s1, Figures S1–S6; Table S1 as well as supporting experimental details.
