# Supplementary material for: Fast Screening of Diol Impurities in Methoxy Poly(Ethylene Glycol)s (mPEG)s by Liquid Chromatography on Monolithic Silica Rods
Source: Polymers (Basel). 2018 Dec 16;10(12):1395. doi: 10.3390/polym10121395 (PMC6401720; doi:10.3390/polym10121395)
Supplement: Supplementary file 1 [file polymers-10-01395-s001.pdf]

Supporting Information

Fast screening of diol impurities in methoxy poly(ethylene glycol)s (mPEG)s by liquid chromatography on monolithic silica rods

Michaela Brunzel,[1,2] Tobias C. Majdanski,[1,2] Jürgen Vitz,[1,2,3] Ivo Nischang*,[1,2] and Ulrich S. Schubert*,[1,2]

[1] Laboratory of Organic and Macromolecular Chemistry (IOMC), Friedrich Schiller University Jena, Humboldtstr. 10, 07743 Jena, Germany.

[2] Jena Center for Soft Matter (JCSM), Friedrich Schiller University Jena, Philosophenweg 7, 07743 Jena, Germany

[3] Center for Sepsis Control and Care (CSCC), Jena University Hospital, Am Klinikum 1, 07747 Jena, Germany

*E-mail: ivo.nischang@uni-jena.de; ulrich.schubert@uni-jena.de

Experimental

Chemicals and materials

Reagents and solvents were commercial products purchased from Aldrich or Linde. Ethylene oxide (EO) was stirred over sodium before distillation into burettes. Prior to use, 2-methoxyethanol and diphenyl methane were stirred over calcium hydride and then distilled under vacuum. The purified reactants were flushed with argon, stored in Schlenk flasks in a glove box, and used within three days. Tetrahydrofuran was purified by heating under reflux over freshly prepared sodium-benzophenone until a deep blue color appeared. The purified tetrahydrofuran was subsequently stored in a Schlenk flask under inert conditions and used within a short time.

HPLC grade acetonitrile was obtained from Sigma (Taufkirchen, Germany) and ultrapure water freshly acquired from a Thermo Scientific™ Barnstead™ GenPure™ xCAD Plus ultrapure water purification system (Thermo Electron LED GmbH, Langenselbold, Germany). The methoxy poly(ethylene glycol) (mPEG) samples utilized in this study were synthesized in-house (vide infra). The PEGdiol samples were obtained as SEC standards from Polymer Standards Service GmbH (PSS, Mainz, Germany) and Polymer Laboratories (PL, Shropshire, UK).

In-house preparation of mPEGs

All preparative steps for obtaining the initiator potassium 2-methoxyethanolate were performed under inert conditions in a glove box. 2-Methoxyethanol was dissolved in tetrahydrofuran and diphenylmethyl potasium (prepared as reported previously[1,2]) was added drop-wise until a precipitation of the product could be observed and a slight orange mixture remained. The product was filtered and washed four times with tetrahydrofuran until the orange color disappeared completely. Afterwards, it was dried under vacuum and appeared as a grey powder.

The preparation of the initiator solutions for the polymerization of EO to mPEG by the living anionic ring-opening polymerization (AROP) was performed in a glovebox. First of all, tetrahydrofuran and potassium 2-methoxymethanolate were added into a GL45 bottle under inert conditions. To

keep the whole process under inert conditions, the fine suspension was then transferred via PTFE tubings into a PicoClave glass autoclave reactor (BüchiGlasUster, Uster, Switzerland) and cooled down to –20 °C under stirring. Afterwards the appropriate amount of EO was added to the reaction mixture by a mini CORI-FLOW mass flow controller device (Bronkhorst High-Tech B.V., Ruurlo, Netherlands), heated successively to 45 °C within 120 min, and stirred for a further 48 h. The polymerization was terminated by the addition of a mixture of ethanol/acetic acid (95/5, % v/v). For isolation and purification, the polymer was precipitated in cold diethyl ether, filtered and dried under vacuum. The product was obtained as a white powder.

Molar mass estimation of in-house prepared mPEGs
Size exclusion chromatography (SEC) measurements were performed on a Shimadzu SEC system (controller: CBM-20A VP, degasser: DGU-20A5, pump: LC-10ADVP, autosampler: SIL-10AD VP, oven: Techlab, RI detector: RID-10A) operated with a ternary mobile phase composed of chloroform/isopropanol/triethylamine 94/2/4 (%, v/v/v) as the eluent. The PSS SDV guard/linear S column (5 µm particle size) was operated with a volumetric flow rate of 1 mL min^{-1} at a temperature of 40 °C. The system was calibrated in a molar mass range of 194 g mol^{-1} up to 106,000 g mol^{-1} with PEG/PEO standards from (i) Polymer Standards Service GmbH (PSS, Mainz, Germany): PEO 106,000; 55,800; 42,700; 26,100 g mol^{-1}, and (ii) Polymer Laboratories (PL, Shropshire, UK): PEG 12,600; 7,100; 4,100; 1,470; 960; 600; 440; 194 g mol^{-1}.

Liquid chromatography
Chromatographic measurements were carried out using an adapted Agilent Technologies 1200 series chromatographic system from Polymer Standards Service GmbH (PSS, Mainz, Germany). Extra-column volumes were minimized by using 130 µm I.D. tubing from the injector to the column head and from the column outlet to the detector. The injection volume was set to 10 µL in all experiments, i.e. 0.6% of the column volume. The column was housed in a TCC 6000 column oven from Polymer Standards Service GmbH (PSS, Mainz, Germany) tempered at 30 °C. A SofTA Model 400 evaporative light scattering detector (ELSD) from Polymer Standards Service GmbH (PSS, Mainz, Germany) was connected to the column outlet tubing. For the ELSD detector, nitrogen was used as carrier gas. The chamber and drift tube temperature were set to 45 and 70 °C, respectively. The detector was operated at the maximum data acquisition rate of 10 Hz. Elutions were performed on a Chromolith® High Resolution RP18 endcapped, reversed-phase monolithic silica rod obtained as a research sample from Merck KgaA (Darmstadt, Germany). The length of the column was 100 mm at a diameter of 4.6 mm. The overall dead volume of the system was measured by replacing the column with a zero-dead volume tube connector and by injection of the smallest and largest PEG sample, respectively. It was calculated to be approximately 2% of the overall column volume.
All samples were prepared at concentrations between 0.1 and 2.0 mg mL^{-1} by dissolving them in the respective mobile phase used for chromatographic experiments. Before analysis, samples were filtered over a 0.45 µm pore size PTFE-Filter.

Matrix-assisted laser desorption ionization-time of flight mass spectrometry (MALDI-TOF-MS)
MALDI-TOF-MS experiments of collected elution fractions were performed with an Ultraflex III TOF/TOF mass spectrometer (Bruker Daltonics, Bremen, Germany) equipped with a Nd-YAG laser. All spectra were measured in the positive reflector mode. The instrument was calibrated prior to each measurement with an external PMMA standard from Polymer Standards Services GmbH (PSS, Mainz, Germany) in the required mass measurement range. MS data were processed using the software Flex Analysis 3.4 and isotope pattern calculator from Bruker Daltonics. For the MALDI-MS sample preparation, the collected elution fractions, a solution of trans-2-[3-(4-*tert*-butylphenyl)-2-methyl-2-propenylidene]malononitrile (DCTB, Sigma Aldrich) in chloroform at a concentration

of 30 mg mL^{-1}, and the doping salt sodium iodide dissolved in chloroform at a concentration of 60 mg mL^{-1}, were used. The elution fraction containing the sample was spotted first on the target plate and the spot was then allowed to dry. After drying, the combined solution of the matrix and the salt were applied to the respective spot on the target plate. For each sample, 0.5 µL of the sample solution was spotted, followed by 0.5 µL of the matrix-salt mixture solutions.

Table S1. Overview of commercial PEGs and SEC results of mPEGs used for chromatographic experiments in this study.

PEG	M_n g mol^{-1}	M_w g mol^{-1}	*Đ*
PEGdiol 1[a]	375	400	1.07
PEGdiol 2[a]	1,840	2,010	1.09
PEGdiol 3[a]	2,800	3,060	1.09
PEGdiol 4[a]	7,500	11,200	1.51
PEGdiol 5[a]	22,100	25,800	1.17
PEGdiol 6[a]	34,000	42,700	1.26
mPEG 1	2,300	2,400	1.04
mPEG 2	5,600	5,800	1.04
mPEG 3	7,500	7,700	1.03
mPEG 4	12,000	13,200	1.10
mPEG 5	21,800	22,700	1.04
mPEG 6	34,900	37,200	1.07
mPEG 7	46,200	50,100	1.08

[a]Properties provided by the manufacturer.

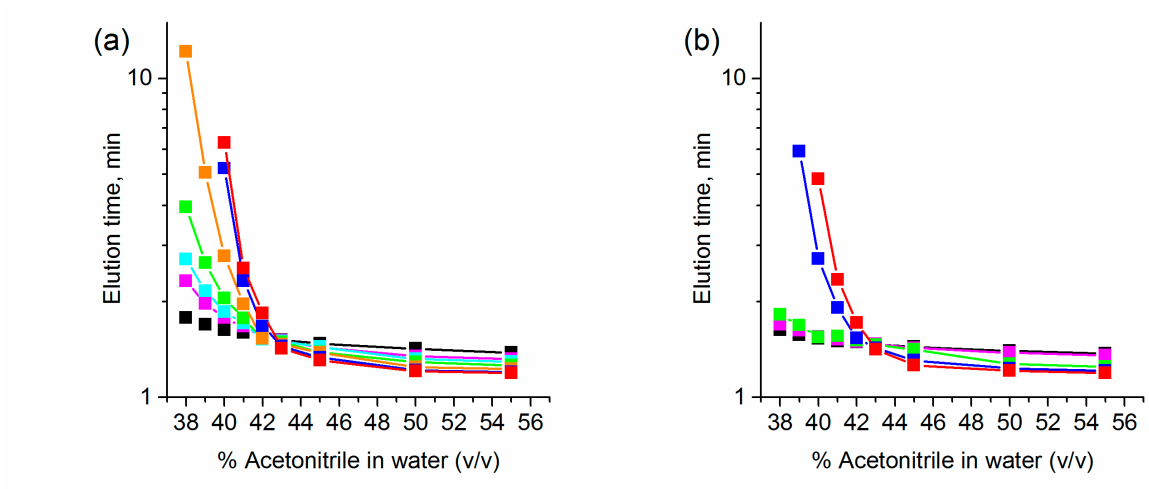

Figure S1. Elution time vs content of acetonitrile in the mobile phase (%, v/v) for (a) mPEGs, and (b) PEGdiols. Colors identifying molar mass of mPEGs in (a): M_n = 2,300 g mol^{-1} (black); M_n = 5,600 g mol^{-1} (magenta); M_n = 7,500 g mol^{-1} (cyan); M_n = 12,000 g mol^{-1} (green); M_n = 21,800 g mol^{-1} (orange); M_n = 34,900 g mol^{-1} (blue); M_n = 46,200 g mol^{-1} (red). Colors identifying molar mass of PEGdiols in (b): M_n = 1,840 g mol^{-1} (black); M_n = 2,800 g mol^{-1} (magenta); M_n = 7,500 g mol^{-1} (green); M_n = 22,100 g mol^{-1} (blue); M_n = 34,000 g mol^{-1} (red).

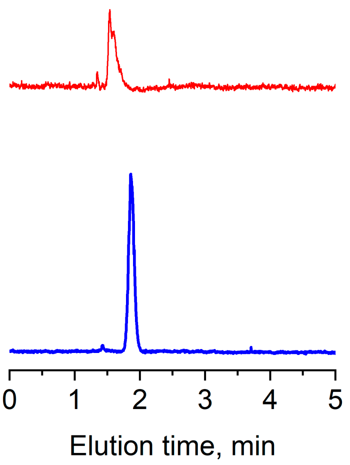

Figure S2. Elugrams of mPEG (M_n = 7,500 g mol^{-1}, blue trace) and PEGdiol (M_n = 7,500 g mol^{-1}, red trace). Mobile phase flow rate of 1 mL min^{-1} and mobile phase composition of 40/60 acetonitrile/water (%, v/v).

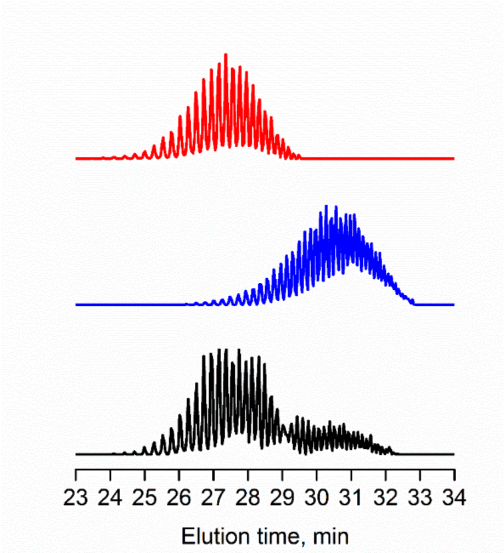

Figure S3. Gradient elution profile of a PEGdiol with M_n = 1,840 g mol^{-1} (red trace), mPEG with M_n = 2,300 g mol^{-1} (blue trace) and their 50/50 (%, v/v) mixture (black trace). Conditions: binary linear mobile phase gradient from 10% acetonitrile in water at an isocratic hold for 3 minutes followed by a linear increase to 50% acetonitrile in water (%, v/v) at 50 minutes.

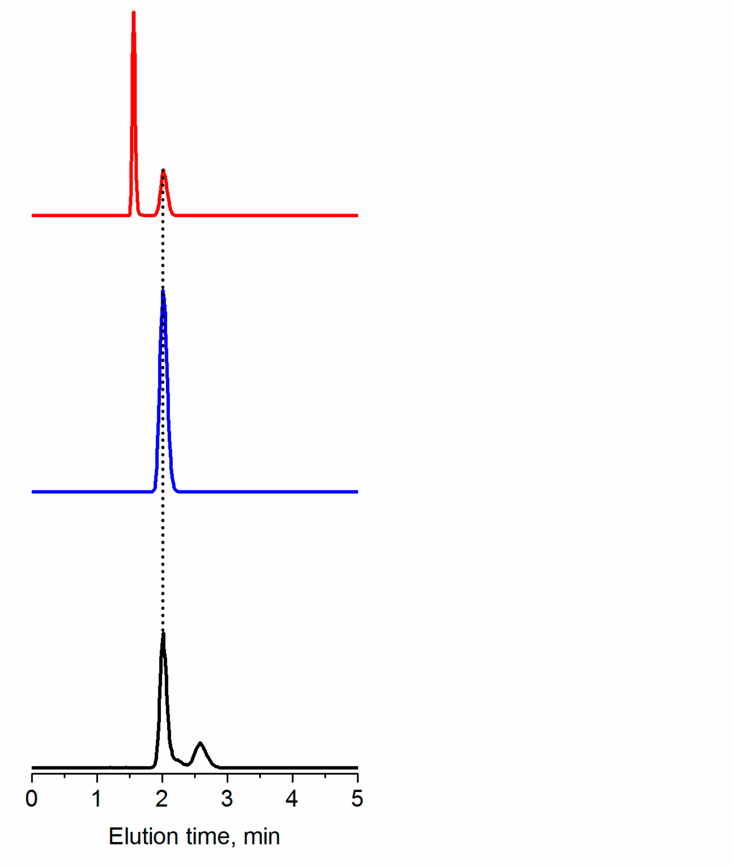

Figure S4. Elugrams of mPEG (M_n = 12,000 g mol^{-1}, blue trace) and 50/50 (%, v/v) mixtures with M_n = 2,800 g mol^{-1} PEGdiol (red trace) and M_n = 22,100 g mol^{-1} PEGdiol (black trace). Mobile phase flow rate of 1 mL min^{-1} and mobile phase composition of 40/60 acetonitrile/water (%, v/v).

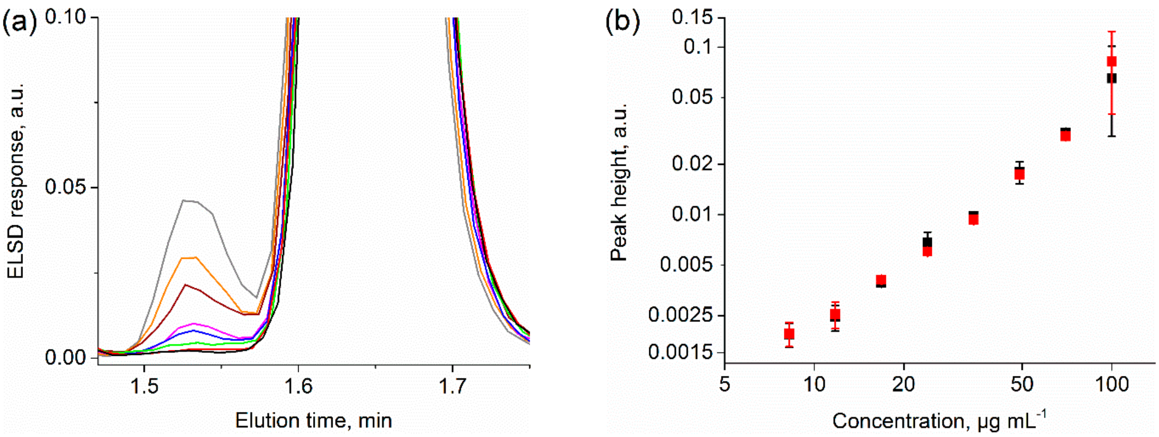

Figure S5. (a) Elution traces of mPEG (M_n = 2,300 g mol^{-1}) held constant at 1 mg mL^{-1} and increased amounts of PEGdiol (M_n = 1,840 g mol^{-1}). (b) Double logarithmic plot of elution signal peak height from (a) against the concentration of PEGdiol in the mixture with error bars from triple injections (black symbols and error bars). The measurements were repeated the next day with identical sample solutions (shown with red symbols and error bars). Mobile phase flow rate of 1 mL min^{-1} and mobile phase composition of 40/60 acetonitrile/water (%, v/v).

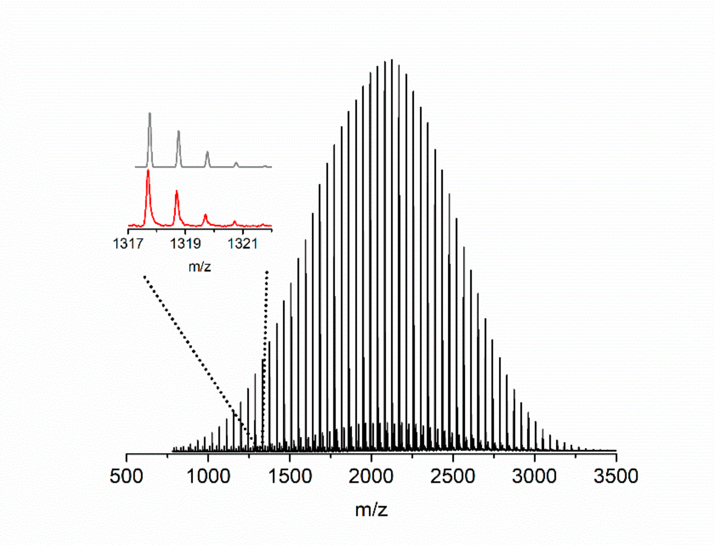

Figure S6. MALDI-TOF-MS of the mPEG intentionally prepared with a protic impurity, i.e. water, added at the start of the presumably living anionic ring-opening polymerization. The inset of a magnified example mass region also contains the isotopic fragmentation pattern of the lower intensity PEGdiol impurity shown in gray.

References

1. Vitz, J.; Majdanski, T.C.; Meier, A.; Lutz, P.J.; Schubert, U.S. Polymerization of ethylene oxide under controlled monomer addition via a mass flow controller for tailor made polyethylene oxides, *Polym. Chem.* **2016**, *7*, 4063-4071, doi:10.1039/C6PY00402D
2. Majdanski, T.C.; Vitz, J.; Meier, A.; Brunzel, M.; Schubert, S.; Nischang, I.; Schubert, U.S. "Green" ethers as solvent alternatives for anionic ring-opening polymerizations of ethylene oxide (EO): In-situ kinetic and advanced characterization studies, Polymer **2018**, *159*, 86-94, doi:10.1016/j.polymer.2018.09.049.
